# Conceptual and methodological concerns in the theory of perceptual load

**DOI:** 10.3389/fpsyg.2013.00522

**Published:** 2013-08-13

**Authors:** Hanna Benoni, Yehoshua Tsal

**Affiliations:** ^1^Department of Psychology, Tel-Aviv UniversityTel-Aviv, Israel; ^2^Department of Psychology, The College of Management Academic StudiesRishon Lezion, Israel

**Keywords:** perceptual load, dilution, early vs. late selection, selective attention, visual attention, distractor interference

## Abstract

The present paper provides a short critical review of the theory of perceptual load. It closely examines the basic tenets and assumptions of the theory and identifies major conceptual and methodological problems that have been largely ignored in the literature. The discussion focuses on problems in the definition of the concept of perceptual load, on the circularity in the characterization and manipulation of perceptual load and the confusion between the concept of perceptual load and its operationalization. The paper also selectively reviews evidence supporting the theory as well as inconsistent evidence which proposed alternative dominant factors influencing the efficacy of attentional selection.

Questions concerning the locus of selective attention have played a central role in the study of attention for decades, (see Johnston and Dark, 1986; Lachter et al., 2004, for reviews). According to early selection views (e.g., Broadbent, 1958; von Wright, 1968; Neisser, 1967; Treisman, 1969; Moran and Desimone, 1985), there is initial, involuntary, parallel processing of the physical characteristics of all stimuli. Based on the information derived from this initial analysis, a stimulus can be selected by attention for further processing to determine its meaning. Thus, unattended stimuli are not processed to the semantic level. On the other hand, late-selection accounts (e.g., Deutsch and Deutsch, 1963; Norman, 1968; Duncan, 1980; Tipper, 1985) hold that selection occurs later in the information processing stream such that there is also an initial, involuntary, parallel semantic processing of all stimuli. Hence, both the physical characteristics and the identities of unattended stimuli are processed.

Based on a distinction initially made by Kahneman and Treisman (1984), Lavie and Tsal (1994; Lavie, 1995) noted a fundamental difference between the two groups of studies described above. While early selection theories have relied on paradigms which were characterized in high display set size (e.g.,visual search paradigms) (for a review, see Pashler and Johnston, 1998), studies which supported the late selection views involved small display set sizes, usually no more than two different items, a target and a distractor (e.g., Eriksen and Eriksen, 1974; Keren et al., 1977; Gatti and Egeth, 1978; Kahneman and Henik, 1981; Hagenaar and van der Heijden, 1986; Miller, 1987; Paquet and Lortie, 1990). On the basis of this critical observation Lavie has developed her hybrid theory of selective attention. According to this theory, processing load of the relevant task determines the extent to which irrelevant distractors are processed. With a low load in relevant processing, leftover resources inevitably spillover to process irrelevant information. The processing of irrelevant distractors could be prevented only when the high load in relevant processing exhausts all attentional resources.

Various studies supported the predictions of perceptual load theory (e.g., Lavie and Cox, 1997; Rees et al., 1997, 2001; Maylor and Lavie, 1998; Lavie and Fox, 2000; Forster and Lavie, 2007, 2008) mostly using display set size for manipulating perceptual load. Thus, in the Low-Load Condition the target appeared by itself in one of several possible positions. In the High-Load Condition the target was embedded among several neutral letters. Distractor interference was measured by the effect of an incongruent relative to a neutral or congruent distractor appearing somewhat remotely from the target. Typically, substantial interference was observed under the Low-Load Condition, but was either markedly reduced or completely eliminated under the High-Load Condition. This finding was interpreted as supportive of load theory (e.g., Lavie, 1995) by assuming that reduced interference under the High-Load Condition is due to the fact that a great deal of attentional resources was required for searching the target among neutral items, leaving no spare resources to be captured by the irrelevant distractor.

Over the past two decades perceptual load theory has received a great deal of attention and has produced considerable impact in the attention literature. The present paper closely examines the basic tenets and assumptions of the theory as well as consistent and inconsistent evidence and identifies major conceptual and methodological flaws in the theory that have been largely ignored in the literature.

## Perceptual load theory is an early, not a hybrid, account of selective attention

The highly acclaimed contribution of perceptual load theory is its hybrid resolution of the early-late selection debate. That is, Perceptual load theory has been presented and recognized as a hybrid model in which the locus of attentional selection is flexible, either early or late, depending on the processing load of the relevant task. However, a closer look suggests that “perceptual load” has been erroneously treated as a hybrid account of attentional selection, since it is exclusively an early selection theory.

The early/late debate can be delineated by two main questions: (1) At which stage in the information processing stream attention selects information? (2). To what extent are unattended stimuli processed (or alternatively, which stages in the information processing stream necessitate attention)? According to perceptual load theory when prioritized relevant processing exhausts all of the available resources, irrelevant information remains unattended and is consequently excluded from processing. Thus, under high load conditions early selection occurs. Unlike the above, in low load presentations the relevant stimuli do not demand all of the available attentional resources, and spare resources unintentionally spill over to irrelevant stimuli, consequently enabling their processing. In other words, the theory actually proposes that the semantic processing of distractors in these displays occurs as a result of attentional allocation over their locations. This proposal is equivalent to the view that attention is necessary for semantic processing, and as such, it is strictly an early selection account. Moreover, since the theory states that the interference in low load conditions is produced by attentional allocation over irrelevant information, and not by preattentive semantic processing of the irrelevant information, the theory actually states that in principle, attention selects relevant information early in processing. Thus, the different patterns obtained under high load and low load presentations, according to the theory, are due to the efficiency of selection: while under high load conditions attentional selection is efficient, it is inefficient under low load conditions. Hence, the theory proposes a single early locus of selection irrespective of perceptual load, but a flexible answer regarding the efficiency of selection.

Why is load theory presented in the literature as a hybrid account concerning the question of the locus of selective attention? This oversight is probably the result of mistaking the efficiency of selection for the locus of selection. Another reason for this confusion could stem from the confusion between the questions of selective attention and selection of relevant from irrelevant information. The role of attention is indeed to select the relevant information, but if attention “spills over” to irrelevant information (as according to load theory, occurs in low load presentations), attentional selection is inefficient. Yet, the observer needs to select the relevant information in order to produce the correct response. Thus, the selection of relevant information from irrelevant will indeed occur late in processing stream, but it is important to realize that this late selection *is not* the *attentional* selection, but rather the decision that observers produce through higher cognitive functions.

In summary, load theory should not be presented as a theory which resolves the early/late debate by suggesting a hybrid model concerning this debate. Instead, load theory deserves its recognition for putting aside the archaic question of the locus of attentional selection, and by shifting the focus of interest to the more adaptive question: the question of the efficiency of selection.

## Alternatives and confounds

Various studies reported inconsistencies with perceptual load theory and proposed that factors other than perceptual load are major determinants of efficient selectivity (e.g., Paquet and Craig, 1997; Fournier et al., 2002; Johnson et al., 2002; Murray and Jones, 2002; Chen, 2003; Theeuwes et al., 2004; Eltiti et al., 2005; Chen and Chan, 2007; Cosman and Vecera, 2012). Below are a few examples. Eltiti et al. (2005) argued that the major factor contributing to effective selection is the *relative salience* of the target and the distractor rather than perceptual load. They jointly manipulated perceptual load and onset (salient) or offset (not salient) of target and distractors. They found effective selection for onset target and offset distractor even under low load conditions, and distractor interference for offset target and onset distractor even under high load conditions. Eltiti et al. (2005) thus concluded that it is salience rather than load that determines the efficacy of selection. Evidence against load theory was also presented by Paquet and Craig (1997) who like Eltiti et al. (2005), showed efficient selectivity under low load conditions. They found that efficient selection heavily depends on *target-distractor similarity* and that distractor interference could occur under low load conditions for near but not far distractors, both for precued and uncued targets.

Chen (2003) showed that increasing perceptual load did not facilitate selection when both the distracting and the target stimuli were part of the same object (see also Kramer and Jacobson, 1991). Cosman and Vecera (2012) further demonstrated that during low-load search, filtering out of the flanker was enhanced when the to-be-ignored letter did not group with the search array (as in the high load search). Conversely, during high-load search, task-irrelevant flanker letters still exerted an interference effect if targets and flankers appeared in the same object (as in the low load search). They proposed that *object based attention effects* play a central role in selective attention regardless of the perceptual load of the task being performed.

Theeuwes et al. (2004) proposed another alternative. They argued that high load and low load conditions differ in *attentional set*. In the former the subjects are engaged in focused attention suitable for a serial search whereas in the latter they employ a distributed mode which is suitable for identifying a single target that can occur in one of several positions. In support of their claim they showed that intermixing high load and low load displays abolish the difference between the two. Consequently, Theeuwes et al. proposed that advance knowledge of perceptual load level rather than perceptual load *per se*, modulates the processing of irrelevant distractors.

Johnson et al. (2002) argue that *mode of attention (focused vs. distributed)* plays an important role in determining distractors interference. They demonstrated efficient selectivity in low load displays when precuing the position of the upcoming target as compared to a no-cue condition. They concluded that the cue in the low load condition helped participants to engage in selective and focused processing. A further interpretation for their findings will be discussed in the next section.

### Dilution vs. perceptual load

Tsal and Benoni (2010a,b; Benoni and Tsal, 2010) have argued that reduction of distractor interference under high load conditions, in set size manipulations, need not be attributed to increases in perceptual load resulting from the need to search for the target among the neutral letters. Instead, it could be due to the dilution of the distractor by the presence of neutral items characterizing high load presentations. These neutral items may play an important role in competing with the distractor for neuronal representation. Indeed, three different studies (Benoni and Tsal, 2010; Tsal and Benoni, 2010a; Wilson et al., 2011) distinguished between the possible effects of perceptual load and dilution by introducing low-load high-dilution displays. These displays contained neutral letters (as in high load conditions) capable of diluting the distractor. Yet, either stimulus or processing requirements allowed for a low load processing mode. For example, in a multiple color display the target color was preknown in the low load—high dilution condition but not in the high load condition (Tsal and Benoni, 2010a). In all experiments using a variety of converging operations distractor processing was either completely eliminated for these new displays (Benoni and Tsal, 2010; Tsal and Benoni, 2010a) or markedly reduced (Wilson et al., 2011) thereby supporting the conclusion that the elimination of distractor interference under the high load condition, repeatedly misattributed to perceptual load, is completely accounted for by dilution. The alternative dilution interpretation received further support from additional subsequent studies (e.g., Dittrich and Stahl, 2011; Marciano and Yeshurun, 2011; Benoni and Tsal, 2012; Benoni et al., in press; but see (Chen and Cave, 2013); Chen and Cave, for the role of attentional focus in dilution). The dilution manipulations produced two additional important findings. First, when the effect of dilution is properly controlled for, contrary to predictions of load theory, a reversed load effect emerges, i.e., it is high perceptual load, not low perceptual load, which produces greater distractor interference (Tsal and Benoni, 2010a; Wilson et al., 2011; Benoni and Tsal, 2012). A similar reversed load effect was reported by Chen and Cave (2013). Second, there is no need to postulate (e.g., Lavie and de Fockert, 2003) that different types of load produce opposite effects of distractor interference. Instead, when dilution is jointly manipulated with perceptual load, sensory degradation and cognitive load (Tsal and Benoni, 2011; Benoni and Tsal, 2012) the results clearly show that when dilution is properly controlled for any increase in task difficulty (be it perceptual, sensory or cognitive) increases distractor interference.

## The problem of circularity and refutation

What is perceptual load? Perceptual load is a vague term that has never been clearly and precisely defined and its consequent operationalizations have been guided primarily by intuitions rather than by a priori rigorous rules. How ought perceptual load be operationally defined? Tsal and Benoni (2010a) proposed that as a hypothetical construct perceptual load needs to be validated by related observables, either by its antecedents, i.e., related stimulus observables or by its consequents, i.e., related response observables. However, it seems that this concept could be verified by neither. Perceptual load could not be validated by rigorous stimulus manipulations since the manipulation of display size as well as the difficulty of perceptual operations are both confounded with sensory and cognitive factors, as will be detailed below. Nor could it be validated by its dependent measure. Overall reaction times (RT) (typically used as a manipulation check for perceptual load) assess overall task difficulty entailing sensory limitations and cognitive demands, and as such could not be used as a pure measure of perceptual load. Thus, the major building block of load theory, perceptual load, is a vague term that has never been clearly and precisely defined.

The lack of a coherent definition of the concept of perceptual load has resulted in circularity in the characterization of load, in the manipulation of load, and in reasoning. In the discussion bellow we will summarize the situations which illustrate this circularity and the consequent problem of refutation (Popper, 1959, 1963).

### Circularity in the characterization of load: is this a high-load or a low-load condition?

Although overall RT is the main measure that could be used to assess perceptual load, in some papers it was completely abandoned as a manipulation check and replaced by the presence or absence of distractor interference itself, which is supposed to be used as a dependent measure for confirming the predictions of perceptual load theory. For example, Lavie and Cox (1997) found that increasing display search size from one to four did not decrease distractor interference although it did increase the overall RT. Distractor interference was reduced only from set size six. Instead of concluding that these data are inconsistent with predictions of load theory, the authors concluded that “as long as the number of items in the relevant display does not exceed capacity, then irrelevant distractors are not rejected from processing” (p. 397). Similarly, Lavie and Robertson (2001), in assessing the effects of perceptual load on neglect, increased display size from two to three items. This manipulation did not increase overall RT but reduced distractor interference by almost 200 msec. Again, in the discussion, the authors considered the two conditions above as low load and high load respectively, although the manipulation check did not confirm any difference in perceptual load. Clearly, the absence of any a priori criteria for changes in perceptual load prevents the theory from standing the refutation criteria (Popper, 1959, 1963).

### Circularity in the manipulation of load: defining load on the basis of stimulus rather than processing considerations

In addition to the circularity associated with the definition of load, there also exists circularity with respect to how load is conceived and operationalized. Consider, for example, a study by Johnson et al. (2002). In this study the authors jointly manipulated display size and cuing. Following Lavie and Cox (1997), they presented a circular configuration containing one target (X or N) and five neutral letters that were flanked by a distractor. In the low load condition the neutral letters were all O's. In the high load condition the neutral letters were heterogeneous and shared features with the targets (and the distractors). Johnson et al. added a cuing manipulation and presented the displays either with no cue or with a 100% valid cue that always pointed to the expected target location. When the cue was absent distractor processing was evident in the low load condition but not in the high load condition, as predicted by perceptual load theory. However, with the valid cue there was no distractor processing even when perceptual load was low. The authors concluded that their results support a weak version of perceptual load theory in showing that while perceptual load is an important factor, it is not the only factor affecting attentional selection.

Table 1 presents a summary of the results obtained by Johnson et al. Most illuminating is condition 4. Given the presence of heterogeneous neutral letters it was inappropriately identified as a “high load” condition. However, the level of perceptual load should be dictated by processing considerations rather than by stimulus considerations. Hence, since the target is cued in advance, the heterogeneous display no longer constitutes a high load condition since the neutral letters need not be actively searched and could be easily filtered out (e.g., Yantis and Johnston, 1990). In further validation of this claim, one can see that the overall RT in this condition is indeed similar to that of the other two low-load conditions and substantially shorter than that of the high-load condition. In fact, this condition is similar to the dilution conditions (characterized by low load and high dilution) used in our previous studies (Benoni and Tsal, 2010, 2012; Tsal and Benoni, 2010a; Benoni et al., in press). Hence, the most important aspect of the results concerns the comparison between conditions 2 and 4 which shows that displays containing heterogeneous neutral letters with cuing (low load) and without cuing (high load), although substantially differing in overall RT (by 275 msec) equally reduced the congruency effect to 11 msec. Therefore, as argued by Tsal and Benoni (2010a); Benoni and Tsal (2010, 2012); Wilson et al. (2011), it is not perceptual load but rather the dilution resulting from the mere presence of neutral interfering letters that reduce or eliminate distractor processing.

**Table 1:** **Mean RTs (average of congruent, neutral and incongruent trials) and congruency effects (incongruent RT—congruent RT) for each condition in Johnson et al.'s (2002) study**.

**Condition**	**Mean RT**	**Congruency effect**
1. No Cue—Low Load	584	65
2. No Cue—High Load	775	11
3. Valid Cue—Low Load	490	8
4. Valid Cue—High Load^*^	500	11

* *This is, in fact, a Low Load condition characterized by low load and high dilution*.

The study of Johnson et al. (2002) illustrates the problem of refutation due to the absence of a coherent definition of the concept of perceptual load. Johnston and his colleagues established their important conclusion on the results obtained from condition 3 (i.e., Valid Cue—Low Load). Since they characterized condition 4 as High-Load (valid cue) condition they hypothesized a-priory a reduction in interference in this condition, although reduction of distractor interference in this condition should undermine the basic tenets of perceptual load theory.

### Circular reasoning: face and emotional distractors under “perceptual load”

Several studies have manipulated load, not in order to test the predictions of load theory, but rather to test whether perception of faces (e.g., Lavie et al., 2003; Reddy and Wilken, 2004) or emotional stimuli (e.g., Bishop et al., 2007; Okon-Singer et al., 2007; Fox et al., 2012) requires attention. These studies have utilized typical load manipulations, but with faces or emotional stimuli used as distractors. The studies are based on the following rationale: If the specific distractor is processed automatically without attention then it is expected to produce interference, irrespective of the level of perceptual load in the task. If, on the other hand, distractor processing requires attention, then perceptual load is expected to reduce distractor interference.

The rationale underlying this line of studies is quite problematic as it produces circular reasoning. The basic assumption of these studies is that the distractors are attended in low load conditions and unattended in high load conditions. The problem is that this assumption cannot be stated a priori since it serves as the *hypothesis* and as the end product of the investigation of load theory, and since load theory uses the *same manipulations* of load, to *test* this assumption (see Lamy et al., 2013, for a related criticism). That is, for example, if manipulating load does not affect distractor interference it may indeed suggest that the processing of this distractor is not affected by attention, or is processed in a specific module. However, this same result can alternatively suggest that this finding is inconsistent with perceptual load theory thereby undermining its very basic assumptions. The latter possibility should not be taken lightly since various studies failed to replicate the traditional load effects (e.g., Theeuwes et al., 2004; Nelson et al., 2012, experiment 2; Tsal and Benoni, 2010a, experiments 2 and 4)., and also because the dilution account, discussed above, suggests that distractor interference does not necessarily depend on attentional resources. This suggestion is in agreement with several studies which find that the “flanker effect” is independent of spatial attentional resources (e.g., Cohen et al., 1995; Ro et al., 2002; Gronau et al., 2009). All these arguments strongly suggest that the notion of automaticity cannot be verified by manipulations of load.

### Vagueness of definitions: can perceptual, cognitive, and sensory loads be truly distinguished?

The expanded theory of load has argued that whereas increased perceptual load reduces irrelevant interference, increased cognitive load (Lavie et al., 2004) and increased sensory load (Lavie and de Fockert, 2003), in fact, produce the opposite effects. Hence, perceptual load needs to be precisely defined so that manipulations of perceptual load could a priori be clearly distinguished from those of cognitive or sensory load so as to rule out any possible bias in assigning a particular load to a particular pattern of results obtained. A close review of the literature suggests that this may be an impossible task because distinctions between these concepts are often fuzzy.

Lavie et al. (2004) defined cognitive load as a form of control which “depends on higher cognitive functions, such as working memory (WM), that are required for actively maintaining current processing priorities to ensure that low-priority stimuli do not gain control of behavior” (p. 339). The problem is that the most commonly used manipulation of perceptual load involves visual search, which cannot purely measure perceptual load because it entails a cognitive component. The operation of searching requires representing the target template, comparing the target template to possible candidate items, and categorizing the items in the search array. Indeed several models have proposed that visual WM is critical for a number of important operations during visual search, (e.g., Duncan and Humphreys, 1989; Bundesen, 1990). Consistent with this view, evidence from several neurophysiological studies have indicated that during visual search neurons that are selective for the search target often remain active during a delay period before the onset of the search display. Interestingly, the same brain areas show template-related activity during the delay period, followed by an enhanced response to a matching target during visual WM tasks (e.g., Miller and Desimone, 1993, 1994). Moreover, cells in inferior temporal (IT) cortex also show enhanced firing rates during search, just before a saccadic eye movement toward that target (Chelazzi et al., 1993). All of these findings led to the conclusion that visual search is just a variant of a WM task. This conclusion is most strongly supported by Luria and Vogel (2011) who tested directly the proposal that perceptual load manipulations by display set size, are actually WM manipulations. They follow the set-size manipulation conducted in Lavie and Cox (1997), and used an electrophysiological measure of WM capacity, the contralateral delay activity (CDA) amplitude, which is a marker for WM capacity (e.g., Vogel and Machizawa, 2004). Luria and Vogel found that the CDA amplitude was larger significantly in high load conditions compared to low load conditions, indicating a greater involvement of WM in the former. As far as the CDA amplitude does indeed reflect WM, then this finding provides direct evidence to the argument that perceptual load manipulations are confounded with memory load manipulations. The argument that perceptual load manipulations are confounded with those of cognitive load can be applied to other load manipulations not involving display size. For example, (Lavie, 1995) (Exp. 2) manipulated perceptual load by different processing requirements for identical displays. In the low load condition participants were required to identify a simple feature (e.g., to press a key only if the figure was red). In the high-load condition, they were required to perform a conjunction task (e.g., to react only if the figure was a red square). Obviously, the high perceptual load condition also imposed greater memory demands as it required two different sets of combinations of features to be held in memory. Moreover, Fournier et al. (2002) have demonstrated that Identification of feature conjunctions does not increase the perceptual demands on attention. Instead, slower responses associated with disjunction-conjunction judgments have been shown to be accounted for by differences in decision activation and/or memory demands (Fournier et al., 2004). Similar confounds of cognitive and perceptual demands are evident in other manipulations of perceptual load such as identifying the letter case vs. counting the number of syllables (Rees et al., 1997).

Manipulations of perceptual load and sensory degradation are also heavily confounded. For example, in the study of Lavie and de Fockert (2003, Exp.3), sensory load was manipulated by reducing visual acuity owing to position eccentricity of the target. This manipulation is confounded with perceptual load, since searching for a more peripheral target is perceptually more difficult irrespective of its reduced acuity. Empirical support for this claim is evident in this same Lavie and de Fockert study which showed a significant interaction between target position and perceptual load. Hence, the mechanisms mediating the effects of sensory and perceptual manipuolations are not independent. In a recent study, Fitousi and Wenger (2011) used more powerful measures as the hazard function of the response time distribution (Townsend and Ashby, 1978; Wenger and Gibson, 2004), along with signal detection theory, to test perceptual load theory. They found that contrary to the assumptions of load theory, perceptual load does, in fact, induce data limitations. Their findings provide strong evidence that perceptual load are confounded with sensory limitations.

The lack of clear distinctions between sensory degradation and perceptual load manipulations have produced confusing results in the literature. In an ERP study Handy and Mangun (2000) found that high perceptual load was associated with a decrease of the P1 and N1 components related to the distractor, supposedly in line with the prediction of load theory. The problem is that the same effects were obtained when perceptual load was manipulated by shortening target duration and superimposing a mask at its location, which are clearly sensory load manipulations. Similarly, in a recent fMRI study Yi et al. (2004) found that increasing the perceptual difficulty of a foveal target task attenuated processing of task-irrelevant background scenes. Again, the problem with this interpretation is that perceptual load was manipulated with sensory degradation, i.e., degrading the central face stimuli with random salt and pepper noise. It seems that the fuzziness of the concept of “perceptual load” permitted the assignment of the results to a particular load which fits the obtained pattern of results.

## Summary

The present paper closely examines the basic tenets and assumptions of the theory of perceptual load and identifies various conceptual and methodological flaws in the theory. The critical discussion focuses primarily on the definition of perceptual load, the difficulty in specifying the nature and level of load, the circularity in the characterization of load and the confusion between the concept of load and its operationalization. Unlike our previous studies (Benoni and Tsal, 2010, 2012; Tsal and Benoni, 2010a,b; Benoni et al., in press), the present paper is not restricted to set size manipulations but rather extends to a general discussion of load theory pertaining to all manipulations of perceptual load.

### Conflict of interest statement

The authors declare that the research was conducted in the absence of any commercial or financial relationships that could be construed as a potential conflict of interest.
